# Fe-C Micro-Electrolysis of HMX: Performance Optimization, Degradation Mechanisms, and Toxicity Evolution Revealed by Toxicogenomics-Based Assay

**DOI:** 10.3390/toxics14060484

**Published:** 2026-05-31

**Authors:** Xin Jiang, Dongqi Wang, Guodong Chai, Guangxiang Duan, Haoting Xiong, Yishi Qian, Lin Xie, Yi Xiao, Heyun Yang, Mingrui Fan, Jiake Li, Yishan Lin, Xiaoliang Li, Yuling Liu

**Affiliations:** 1 State Key Laboratory of Water Engineering Ecology and Environment in Arid Area, Xi’an University of Technology, Xi’an 710048, China; 2 Department of Municipal and Environmental Engineering, School of Eco-Environmental & Chemical Engineering, Xi’an University of Technology, Xi’an 710048, Chinaxhaoting2026@163.com (H.X.);; 3 Shaanxi Key Laboratory of Water Resources and Environment, Xi’an University of Technology, Xi’an 710048, China; 4 China Coal Technology & Engineering Group (CCTEG) Xi’an Research Institute (Group) Co., Ltd., Xi’an 710077, China; 5 Xi’an Modern Chemistry Research Institute, Xi’an 710065, China; 6 School of Public Health and Health Management, Henan Medical College, Zhengzhou 451191, China; 7 Shaanxi Key Laboratory of Earth Surface System and Environmental Carrying Capacity, College of Urban and Environmental Sciences, Northwest University, Xi’an 710127, China

**Keywords:** micro-electrolysis, HMX, performance optimization, *E. coli* cell array assay, toxic effects

## Abstract

This study evaluated the degradation of 1,3,5,7-tetranitro-1,3,5,7-tetrazocane (HMX) in simulated wastewater using an iron-carbon (Fe-C) micro-electrolysis system. The treatment efficiency was systematically evaluated under varying initial pH, Fe dosage, and Fe/C mass ratios. Under the optimized operating conditions (initial pH of 4, Fe dosage of 70 g/L, and an Fe/C mass rat of 1:1), the system achieved a maximum HMX removal efficiency of 98.4%. Kinetic analysis indicated that the degradation process conformed to pseudo-first-order kinetics. Mechanistically, HMX removal was attributed to interfacial adsorption and co-precipitation via in situ generated Fe^2+^ and Fe^3+^ hydroxides, alongside reductive transformation mediated by Fe, Fe^2+^, and nascent hydrogen ([H]) evolved during the micro-electrolysis process. To assess the molecular toxicity evolution of the treated wastewater, a toxicogenomic assay was deployed to evaluate the molecular toxicity evolution of the treated wastewater matrix. The transcriptomic profiling revealed that DNA damage and oxidative stress were the predominant cellular stress responses induced by the wastewater. While the total toxic effect transcript index (TELI_total_) exhibited a transient initial increase before steadily declining, the overall toxic potency remained within a relatively stable range throughout the treatment cycle. Ultimately, this study provides critical insights into process optimization and pathway elucidation, demonstrating that Fe-C micro-electrolysis is a promising and scalable pretreatment technology for the remediation of energetic compound-laden industrial effluents.

## 1. Introduction

With the increasing emphasis on national security worldwide, the rapid development of modern weapon systems has led to increasingly stringent requirements for the properties of energetic materials, especially the urgent need for insensitive high-energy materials with both ‘high energy’ and ‘high safety’ [1]. Energetic materials mainly refer to the metastable materials such as explosives, propellants, pyrotechnics, and flares that can quickly release a large amount of energy without external oxidants [2,3]. HMX, also known as cyclotetramethylene tetranitramine, is a white crystalline high explosive. It is currently one of the most widely used single-component explosives because of its high energy density and excellent overall performance, and is extensively applied in plastic explosives, nuclear devices, rocket fuel and other fields [4]. However, the process of producing HMX is inevitably accompanied by the generation of wastewater pollution, containing large quantities of toxic and hazardous pollutants. The pollutants of wastewater are characterized by stable chemical properties and are difficult to degrade by general microorganisms, which can seriously threaten the ecological balance as well as the health and survival of human being if they are directly discharged [5,6]. Therefore, how to remove HMX wastewater effectively and stably has been the focus of attention.

Iron–carbon (Fe-C) micro-electrolysis is an advanced wastewater treatment process that uses iron as the anode and carbon as the cathode. A galvanic cell is formed due to the potential difference between the two electrodes, thereby inducing redox reactions on their surfaces. This method can destroy the chain of macromolecular organic matter or cyclic organic matter, and improve the biodegradability of wastewater [7]. The main reaction mechanisms are as follows [8,9]:

Anode: Fe − 2e^−^ → Fe^2+^ E_0_ (Fe^2+^/Fe) = −0.44 V (1)

Cathode: 2H^+^ + 2e^−^→2[H]     E_0_ (H^+^/[H]) = 0.00 V (2)

When the cathode is under aerated conditions, the main reaction mechanism is: (acid conditions)

O_2_ + 4H^+^ + 4e^−^ → 2O· + 4[H] → 2H_2_O   E_0_ (O_2_/H_2_O) = +1.23 V (3)

Fe^2+^ + H_2_O_2_ → Fe^3+^ + ·OH + OH^−^ (4)

(Neutral or alkaline conditions)

O_2_ + 2H_2_O + 4e^−^ → 4OH^−^  E_0_ (O_2_/OH^−^) = +0.40 V (5)

Under acidic and aeration conditions, the cathode of the Fe-C system accepts electrons and produces a large quantity of highly reactive [H], thereby realizing the removal organic pollutants in wastewater while simultaneously generating H_2_O_2_ [10]. In addition, Fe^2+^ reacts with the generated H_2_O_2_ through a Fenton-like reaction, generating hydroxyl radicals (·OH) with strong oxidative capacity, which are among the strongest oxidizing species after fluorine [11]. The hydroxyl radicals can effectively destroy the carbon chains of persistent organic pollutants (POPs) ultimately mineralizing them into CO_2_, H_2_O, and inorganic ions. Therefore, the Fe-C micro-electrolysis system has become an important research focus in the treatment of refractory organic pollutants. The factors affecting the pollutant removal efficiency in micro-electrolysis include Fe/C mass ratio, pH, filler dosage and aeration intensity [12,13]. Thus, it is necessary to optimize the degradation conditions to achieve the optimal reaction efficiency. In addition, the degradation of HMX generates multiple intermediate products and involves complex reaction pathways [14,15].

The toxicity of HMX plays an important role in its biodegradation and environmental transformation. If the HMX content in the environment exceeds the microbial tolerance concentration, it will affect the activity of the microbial community. Gou et al. [16] developed a toxicogenomics-based biological effects testing platform to reflect the biological pathway responses at the early stage of the toxicogenic process of chemicals. This method is fast, sensitive, and low-cost, and can screen and evaluate the toxic effects of existing compounds in large quantities, which not only helps deepen the researchers’ understanding of the mechanism of toxicity, but also helps to discover biomarkers of chemical toxicity and exposure. Therefore, the toxicogenomics assay can be used to study the toxic mechanism of HMX and its degradation by-products and provide a scientific basis for its health risk assessment and prevention and control measures.

Based on previous research findings and existing limitations, this study was conducted to investigate the degradation of HMX in wastewater by using Fe-C micro-electrolysis. The optimal process conditions and reaction mechanisms were systematically explored to provide fundamental data and guidance for the large-scale application of HMX wastewater treatment. In addition, toxicogenomics analysis was employed to investigate the toxicity mechanisms of HMX and its degradation by-products, thereby providing a scientific basis for health risk assessment.

## 2. Materials and Methods

### 2.1. Experimental Design

HMX was supplied by Gansu Yinguang Chemical Industry Group Co., Ltd.(Baiyin, Gansu, China). Its purity, melting point, and moisture/volatile content were analyzed by Xi’an Modern Chemistry Research Institute. The results showed that the HMX purity was 99.9%, the melting point was 273.7 °C, and the moisture and volatile matter content was 0.02%, according to the test method specified in GJB2335A-2019 [17]. As shown in Figure S1, a total of 10 mg HMX was dissolved in 500 mL deionized water and stirred at 45 °C to obtain 20 mg/L HMX wastewater. The iron chips were purchased from Ningjin Shuoli Machinery Co., Ltd. (Dezhou, Shandong, China). Due to impurities, such as oxide films and oil stains on the iron chips surface, direct utilization adversely affects the treatment efficiency of the iron-carbon micro-electrolysis process; therefore, activation pretreatment is required. The iron chips with a diameter of 3–5 mm was immersed in a 20% NaOH solution for 2 h to remove surface oil stains and rinsed until neutral. Subsequently, it was soaked in a 10% HCl solution for 2 h to eliminate surface oxides and enhance its reducing capability, followed by rinsing until neutral and drying for future use. The wood-based columnar activated carbon, with a diameter of approximately 2–4 mm, was purchased from Luoyang Bailian Environmental Protection Technology Co., Ltd. (Luoyang, Henan, China).

The diluted H_2_SO_4_ (1 mol/L) and NaOH (1 mol/L) were used to adjust the pH of HMX wastewater. Different Fe-C micro-electrolysis systems with HMX as the target pollutant were established, and batch experiments were carried out. In each batch experiments, 500 mL of HMX wastewater and the required amount of iron chips and activated carbon were added to a 1000 mL beaker, and the reaction was kept at a constant temperature of 45 °C during the experiment. This study was conducted under atmospheric pressure without sealing or external aeration, where dissolved oxygen (DO) was supplied solely through natural contact with the ambient air. To ensure uniform mixing and minimize external mass transfer resistance, all experiments were carried out under continuous magnetic stirring at a constant speed of 200 rpm. During the experiment, samples were taken at different time periods (0 min, 15 min, 30 min, 45 min, 60 min, 90 min, 120 min, and 150 min). In this study, single-factor experiments were primarily conducted to evaluate the effects of initial pH, iron dosage, and Fe/C mass ratio on the treatment efficiency of HMX wastewater. The experimental conditions investigated included a pH range of 2.0–7.0, iron dosages of 10, 20, 30, 40, 50, and 70 g/L, and Fe/C mass ratios of 0.5, 0.6, 0.8, 0.9, 1.0, 1.1, 1.2, 1.3, and 1.4. The specific single-factor experimental parameters were shown in Table S1. All test solutions were collected from the supernatant, passed through a 0.45 μm filter, and stored in vials until HPLC analysis. After determining the optimal experimental conditions, HMX wastewater at different time periods was taken for subsequent toxicological analysis.

### 2.2. Analytical Methods

#### 2.2.1. Chemical Analysis

The concentration of HMX solution was determined by HPLC (PerkinElme, USA). The detection wavelength was 236 nm. The mobile phase was 40% acetonitrile and 60% water. The C18 chromatographic column (column temperature: 35 °C; flow rate: 0.7 mL/min; injection volume: 20 μL) was used.

The degradation products of HMX were further analyzed using LC-MS/MS. The mass spectrometer (U3000, Thermo Fisher Scientific, Waltham, MA, USA) was equipped with an electrospray ionization (ESI) source and operated in positive ESI mode.

#### 2.2.2. Toxicity Analysis and Calculation

Toxicity analysis of HMX wastewater was performed using a toxicogenomic assay adapted from previous protocols. A library of 114 transcriptional fusions in Escherichia coli K12 MG1655 was employed, featuring distinct promoters that regulate green fluorescent protein (GFP) expression across five major stress response pathways: oxidative, DNA, protein, membrane, and general stress (Table S2). Briefly, E. coli cells were cultured in 1× M9 medium using clear-bottom black 384-well plates (Costar, Bethesda, MD, USA) at 37 °C for 5–6 h to achieve early exponential growth (OD600 ≈ 0.2). Subsequently, 10 μL of HMX samples collected at varying degradation intervals were inoculated into each well. A microplate reader (Cytation 5, Bio-Tek, Winooski, VT, USA) was utilized to simultaneously monitor OD600 for cell growth and GFP intensities (excitation: 485 nm; emission: 528 nm) every 5 min over a 2 h period. All assays were conducted in triplicate under dark conditions. It is worth noting that the E. coli-based assay was conducted as a genotoxicity evaluation to investigate transcriptional responses of stress-related biomarkers under HMX wastewater exposure, rather than to assess bacterial removal, growth inhibition, or morphological changes.

All data were corrected by blank medium and promoterless bacterial controls (with and without toxicants), respectively. The gene expression alteration was called induction factor I (I = Pe/Pc), where Pe = (GFP/OD) experiment and Pc = (GFP/OD) control. Then, the natural log of the I value (ln(I)) at every time point is compiled for further analysis [18]. The toxicity values of chemicals were quantified by the TELI, and TELI values represent the magnitude of altered gene expression for each gene response to toxicant exposure for 2 h (a test chemical is defined as toxic when the TELI value is >1.5) [19]. The calculation method uses the following equation [18]:

(6) $${\mathrm{TELI}}_{\mathrm{gene}}\;=\;\frac{\int_{t=0}^{t}\left(e^{\left|\mathrm{lnI}\right|}\;-\;e^{\left|\ln1\right|}\right)\mathrm{dt}}{\mathrm{Exposure}\;\mathrm{Time}}$$

(7) $${\mathrm{TELI}}_{\mathrm{stress}}\;=\;\frac{\sum_{i=1}^{n}\mathrm{wi}\;\times \;({\mathrm{TELI}}_{\mathrm{genei}})}{n}$$

(8) $${\mathrm{TELI}}_{\mathrm{total}}\;=\;\frac{\sum_{j=1}^{n}\mathrm{wj}\;\times \;({\mathrm{TELI}}_{\mathrm{pathwayj}})}{n}$$

where t (h) is the exposure time; i and j are the number of genes/stress pathway in the assay library; w_i_ and w_j_ are the weighting factors for the gene (i) and pathway (j); and in this study, all weighting factors were assigned a value of 1.

The stress genes selected are showed in Table S2.

### 2.3. Data Processing

Scatter plots for this study were generated using Origin 2021 (OriginLab Corporation, Northampton, MA, USA). Biomarker gene summary table were drawn using powerpoint2016 (Microsoft Corporation, Redmond, WA, USA).

## 3. Results and Discussion

### 3.1. Effect of pH

**Figure 1:**
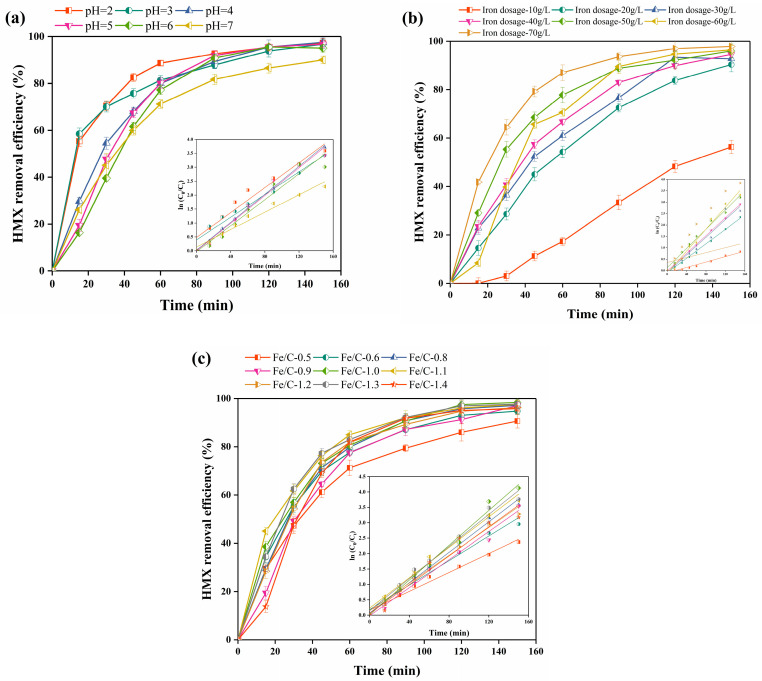
Effect of Fe-C micro-electrolysis on HMX removal efficiency under different conditions (**a**) pH value; (**b**) iron dosage and (**c**) Fe/C mass ratio.

In the Fe-C micro-electrolysis system, initial pH is a key factor affecting the removal of organic matter [7]. It can be seen from Equations (1)–(5) that when the pH of the Fe-C system is low, the potential of oxygen increases, leading to an increase in the potential difference of the reaction system, which promotes the micro-electrolysis reaction to produce more O and [H], thus improving the removal efficiency of organic pollutants [20,21]. As shown in Figure 1a, when the initial pH was 2, the removal efficiency of HMX was 97.2%. With the increase in pH, the removal efficiency slightly increased. When the initial pH reached 4, the removal efficiency reached a maximum of 97.6%. HMX is highly stable in water, meaning it breaks down very slowly and can persist in aquatic environments and migrate into groundwater. When the pH was increased from 2.0 to 4.0, the HMX removal efficiency exhibited a negligible enhancement of only 0.4%, indicating that the micro-electrolysis performance was insensitive to pH fluctuations within this specific range. This phenomenon can be primarily attributed to the intrinsic chemical inertness of HMX and its highly symmetrical cyclic nitramine structure of HMX, which poses a high energy barrier for structural cleavage under current conditions [22]. Furthermore, the elevation of pH to 4.0 might exert dual competitive effects: while it suppresses the parasitic hydrogen evolution reaction [7], it simultaneously reduces the electrochemical driving force due to the decreased H^+^ concentration [23]. The balance between these two competing effects ultimately resulted in a plateaued removal efficiency.

In addition, the inset in Figure 1 shows the linear regression of HMX removal with time during Fe-C micro-electrolysis degradation, where ln(C_0_/C_t_) increased linearly with time, confirming that HMX degradation during micro-electrolysis degradation followed pseudo-first-order kinetics. When the initial pH of the Fe-C system was low (2 or 3), there was an excessive concentration of H^+^, which will preferentially react with Fe, thereby suppressing the formation of ferrous hydroxide and ferric hydroxide flocs. This, in turn, increases the consumption of iron and weakens the galvanic cell reaction, which is not unfavorable for the degradation of organic matter in wastewater [12]. Moreover, Fe may be consumed by corrosion under strong acid conditions. Previous studies have shown that when initial pH < 3, a large amount of H_2_ formed on the surface of Fe, hindering electron transfer and Fe^2+^ release [7,8,24]. When the pH was further increased to 5, the removal rate of HMX decreased slightly to 96.7%. When the initial pH of Fe-C system was increased to 7, the HMX removal efficiency decreased to a minimum of 90.0%. With the increase in pH, the OH^−^ in the solution increased subsequently, which promoted the formation of Fe(OH)_2_ and Fe(OH)_3_. However, due to the increase in OH^−^, the concentration of H^+^ decreases, resulting in a decrease in oxygen potential difference and galvanic cell reaction rate, and the formation rate of the anode Fe^2+^ decreases [7]. In addition, the generation of Fe^2+^ can react with OH^−^ to form Fe(OH)_2_ covering the surface of iron and carbon, further hindering the galvanic cell reaction and greatly affecting treatment performance, which makes the removal efficiency of HMX suboptimal [10]. In summary, the initial pH of 4.0 was the optimal reaction condition for HMX degradation in this study.

### 3.2. Effect of Fe Dosage

The Fe dosage is an important parameter affecting the removal of organic pollutants. As shown in Figure 1b, when the Fe dosage was 10 g/L, the HMX removal efficiency was 56.3%. As the Fe dosage increased, the treatment performance gradually improved, and the HMX removal efficiency reached 90.3% at 20 g/L. At lower Fe dosages, the reduced HMX removal efficiency may be associated with fewer effective Fe-C contact sites and weaker micro-electrolysis reactions. As the Fe dosage increased to 70 g/L, the HMX removal efficiency reached the highest observed value of 97.8%. However, compared with the lower dosage intervals, the removal efficiency increased only slightly from 96.0% to 97.8% as the Fe dosage increased from 50 g/L to 70 g/L. This suggests that the system was approaching a performance plateau within the tested Fe dosage range, and further increases in Fe dosage may provide limited improvement in treatment efficiency.

It should also be noted that excessive Fe dosage may reduce the effective contact between Fe-C particles and wastewater, which could negatively affect treatment performance [25]. In addition, higher Fe and carbon dosages may increase the production of iron sludge, thereby increasing subsequent treatment costs. Under the tested conditions, the highest HMX removal efficiency was achieved at Fe dosage of 70 g/L, without observable deterioration in pollutant degradation performance. Therefore, 70 g/L was selected as the Fe dosage for subsequent experiments.

### 3.3. Effect of Fe/C Mass Ratio

In the Fe-C micro-electrolysis system, iron provides electrons to facilitate the removal of POPs. Meanwhile, the Fe/C mass ratio affected the formation of the galvanic cell in the system. When the Fe/C mass ratio was appropriate, the contact area can be increased and formed more galvanic cells, which facilitated the removal of POPs [10,12,26]. As shown in Figure 1c, when the Fe/C mass ratio was 0.5, the removal efficiency was the lowest, at 90.7%. As the Fe/C mass ratio increased, the removal efficiency of HMX gradually increased. When the Fe/C mass ratio reached 1.0, the HMX removal efficiency reached its maximum value of 98.4%. However, with a further increase in the Fe/C mass ratio, the treatment performance declined. It can be seen from Equation (1) that when the iron content was much lower than that of carbon, the anode iron in the Fe-C system could not provide sufficient electrons to sustain redox reactions on the electrode surface, thereby reducing wastewater treatment efficiency. When the Fe/C mass ratio increased to a certain value, it promoted to the formation of galvanic cells and electrode reaction, which promotes the degradation of organic matter and achieves wastewater treatment efficiency [10]. Accordingly, it was concluded that an Fe/C mass ratio of 1:1 was the optimal condition for HMX removal in the Fe-C micro-electrolysis system. It should be noted that while the current batch experiments demonstrated high efficiency in HMX degradation, the long-term stability and reusability tests of the Fe-C micro-electrolysis material were not performed at this moment. These aspects are critical for practical applications and will be systematically investigated in our future work to evaluate the material lifespan and passivation mechanisms.

### 3.4. Prediction of the Degradation Pathway of HMX

**Figure 2:**
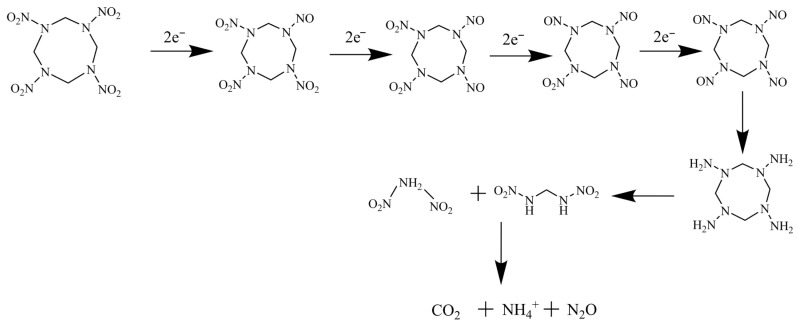
Possible pathways for Fe-C micro-electrolysis degradation of HMX.

The intermediate products of Fe-C micro-electrolysis degradation of HMX were analyzed by LC-MS/MS. Table S3 and Figure S2 show the MS results of HMX intermediates produced by the micro-electrolysis system within 150 min. Based on these results, the possible degradation pathways were determined (Figure 2). Five intermediate products were produced in the whole process. According to LC-MS/MS (ESI^+^) analysis, a sequential mass decrease of 16 Da was observed among the detected intermediate products, suggesting the stepwise conversion of nitro groups (−NO_2_) to corresponding nitroso derivatives (−NO). This characteristic molecular weight shift is consistent with the reductive deoxygenation pathway promoted by the Fe-C micro-electrolysis system. Mechanistically, iron dissolution at the microscopic galvanic anodes liberates a continuous flux of electrons, which synergistically couple with the highly active nascent hydrogen [H] generated at the cathodes, may have contributed to the reductive transformation of the electron-deficient nitro groups on the HMX ring. As a result, four sequential nitroso derivatives were tentatively identified, including octahydro-1-nitroso-3,5,7-trinitro-1,3,5,7-tetrazocine (1NO-HMX, II), octahydro-1,3-dinitroso-5,7-dinitro-1,3,5,7-tetrazocine (2NO-HMX, III), octahydro-1,3,5-trinitroso-7-nitro-1,3,5,7-tetrazocine (3NO-HMX, IV), and octahydro-1,3,5,7-tetranitroso-1,3,5,7-tetrazocine (4NO-HMX, V). These observations support that reductive deoxygenation was an important transformation pathway during Fe-C micro-electrolysis treatment. However, due to the lack of reagents, these intermediates cannot be quantified. As the reaction continues, the resulting [H] undergoes a ring cleavage reaction with the nitroso derivative, followed by the formation of other intermediates, such as methylenedinitramine. These by-products can be continuously oxidized, accompanied by the formation of CO_2_/N_2_O and CH_4_. Furthermore, HMX can be removed by hydroxides of Fe^2+^ and Fe^3+^ by adsorption co-precipitation, etc., while being degraded by the reduction of Fe^2+^ and [H] generated by the micro-electrolysis process.

### 3.5. Toxicity Analysis of HMX Degradation Process by Fe/C Micro-Electrolysis

#### 3.5.1. Changes of TELI in Stress Pathways

A total of 114 marker genes were examined for stress effects in this study, and since the respective changes in the genes were extremely complex, the responses of the five major categories (DNA stress, oxidative stress, protein stress, membrane stress, and general stress) were summarized and analyzed at different response times. As shown in Figure S3, the HMX degradation reaction solution was found to induce strong oxidative and general stress responses. As the degradation time increased, the TELI_total_ values showed an overall trend of increasing and then decreasing, although the overall differences were not significant. However, TELI_total_ was larger than 1.5 for the reaction time of 150 min, indicating that the reaction system was continuously toxic.

#### 3.5.2. Toxicity Mechanism on Molecular Level

**Figure 3:**
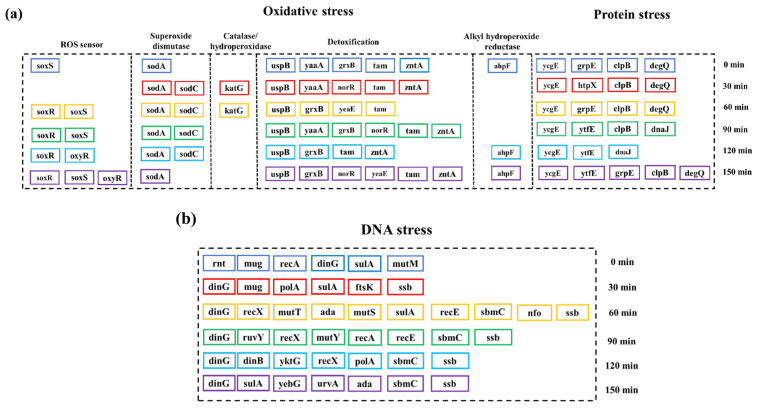
Altered expression of major stress response pathways and biomarker genes during Fe-C micro-electrolysis of HMX (TELI_genes_ > 1.5); (**a**) oxidative stress and protein stress; (**b**) DNA stress.

In order to better understand the mechanism of toxic effects during the removal of HMX by Fe-C micro-electrolysis, 114 marker genes were further analyzed. As shown in Figure 3a, as HMX was degraded, the reaction stages at different treatment periods caused dysregulation of several functional genes in tested E. coli cells.

The number of genes showing altered expression during HMX degradation (Figure 3a) increased from 12 at the initial stage to 13 at 60 min and further increased to 16 at 180 min. For oxidative stress, the Fe-C micro-electrolysis system produced H_2_O_2_ under aerobic conditions, which subsequently catalyzed H_2_O_2_ to generate ·OH radicals with strong oxidation. Therefore, three reactive oxygen species (ROS) sensor genes, namely *sox*R, *sox*S, and *oxy*R, were identified. Enzymes encoded by these genes can scavenge oxidative free radicals to protect cells from harm [27]. For example, *sox*R proteins have a negative autoregulatory function that controls the ability to participate in defense against redox [28]. Furthermore, *sox*R may also be involved in resistance to some antibiotics [29].

Dysregulation of *oxy*R was observed at 150 min during HMX degradation, indicating its involvement in the regulation of cellular peroxide metabolism, redox balance and peroxide protection [30]. *Sox*S is involved in the scavenging of superoxide and protects organisms from organic solvents and antibiotics [29]. Two superoxide dismutase (SOD) enzymes, *sod*A and *sod*C, are the first line of defense against ROS, converting superoxide radicals into hydrogen peroxide and water [31]. It was also found that an alkyl hydroperoxide reductase (*ahp*F) showed dysregulation in the reaction stage (30–60 min), which may be caused by the production of H_2_O_2_ during the micro-electrolysis process. The main function of the hydrogen peroxide reductase encoded by the gene *ahp*F is to scavenge endogenously produced organic hydroperoxides and H_2_O_2_ [32]. We also found that the catalase/hydroperoxidase gene *ka*tG was dysregulated from 30 to 60 min, indicating that high concentrations of H_2_O_2_ may be cleared [33]. Additionally, biomarkers involved in the detoxification pathway also showed dysregulation. For example, *yaa*A, which is associated with cellular physiological responses to hydrogen peroxide stress, has a dysregulation from 0 to 90 min, and the expression of *yaa*A is regulated by *oxy*R when the concentration of H_2_O_2_ is too high. Anne Farewell et al. [34] found that the presence of H_2_O_2_ causes oxidative stress response in cells, leading to excessive expression of the gene *usp*B.

For the DNA stress pathway, seven of the 20 genes showed altered expression dur-ing HMX degradation (Figure 3b). Two of the genes involved in base excision repair (BER) were dysregulated, *mut*T and *nfo* [35]. Gene *ada* is one of two independent direct repair mechanisms in E.coli that control the transcription of genes involved in the repair process of alkylated DNA [27]. Dysregulation of *fts*K was only observed at 30 min during HMX degradation. *fts*K, an important cytokinesis protein that connects cell division to chromosome segregation [36], suggests that the Fe-C micro-electrolysis process inhibits cell division. In addition, DNA stress showed a sustained up-regulated expression over the 60 min response time. In response to these categories of damage, the gene is involved in intracellular SOS regulation, which repairs DNA and prevents the damage from intensifying. Genes involved in DNA repair, such as *rec*E, *rec*A, *uvr*A, *yeb*G, *ssb,* and *sbm*C, were up-regulated in treatment system. Among them, *rec*A, and *rec*E control the transcription of cell response genes related to DNA damage and participate in SOS regulation [37]. The encoded protein of *sbm*C inhibits the binding of DNA to gyrase, which may protect cells from DNA damage caused by gyrase via DNA binding [38]. The *ssb* gene is a highly stable single-stranded DNA-binding protein that plays a central role in DNA replication, recombination, and repair [39]. Nucleotide excision repair is a widespread DNA repair process that can repair a wide range of DNA damage. The *uvr*A gene is indicative of this repair pathway [40].

## 4. Conclusions

The Fe-C micro-electrolysis process exhibits superior capability for the treatment of HMX-containing wastewater, where system performance is strongly influenced by initial acidity, Fe dosage, and the Fe/C mass ratio. The reaction progression exhibits excellent agreement with the pseudo-first-order kinetic model, indicating that the degradation rate is predominantly controlled by the available reactive surface sites and initial contaminant concentrations. The results of genetic toxicology showed that the solution of HMX degradation reaction would produce high oxidative stress and general stress response. With the passage of reaction time, TELI_total_ showed a first increasing and then decreasing tendency, with no significant fluctuation during the whole process. However, the reaction system was continuously toxic (TELI_total_ > 1.5). The damage repair pathways that respond at different periods include SOS response/DNA repair, oxidative stress, detoxification, protein stress, drug resistance/sensitivity, general function, general stress, killer cells, and cold shock. Among them, the Fe-C system will cause a strong and sustained response to cellular oxidative stress while producing H_2_O_2_ and ·OH. As treatment time continued, the number of up-regulated gene expression showed an overall upward trend, indicating that the degradation of HMX in the Fe-C system was accompanied by an increase in toxicity. Therefore, in the future, it is possible to study the toxic mechanism and magnitude of different degradation products of HMX, so as to provide scientific basis for its health risk assessment and prevention and control measures.

## Data Availability

The original contributions presented in this study are included in the article.

## Supplementary Materials

The following supporting information can be downloaded at: https://www.mdpi.com/article/10.3390/toxics14060484/s1, Figure S1: Iron-carbon micro-electrolysis experimental setup diagram; Figure S2: Ion spectrum of HMX degradation intermediate products in positive ion mode; Figure S3: Changes in TELI-based toxicity profiles during Fe-C micro-electrolysis of HMX; Table S1: Single-factor experimental design; Table S2: Stress gene library and its main functions; Table S3: Main degradation intermediates of HMX.
