# Supplementary material for: Fe-C Micro-Electrolysis of HMX: Performance Optimization, Degradation Mechanisms, and Toxicity Evolution Revealed by Toxicogenomics-Based Assay
Source: Toxics. 2026 May 31;14(6):484. doi: 10.3390/toxics14060484 (PMC13306800; doi:10.3390/toxics14060484)
Supplement: Supplementary file 1 [file toxics-14-00484-s001.zip › toxics-4295366-supplementary.pdf]

Fe-C Micro-Electrolysis of HMX: Performance Optimization, Degradation Mechanisms, and Toxicity Evolution Revealed by Toxicogenomics-Based Assay

Xin Jiang [a,b], Dongqi Wang [a,b,c,d*], Guodong Chai [a,b,e], Guangxiang Duan [b], Haoting Xiong [b], Yishi Qian [f], Lin Xie [b], Yi Xiao [b], Heyun Yang [b], Mingrui Fan [g], Jiake Li [a,b], Yishan Lin [h], Xiaoliang Li [a,b], Yuling Liu [a,b*],

[a] State Key Laboratory of Water Engineering Ecology and Environment in Arid Area, Xi'an University of Technology, Xi'an, Shaanxi 710048, China

[b] Department of Municipal and Environmental Engineering, School of Water Resources and Hydro-Electric Engineering, Xi'an University of Technology, Xi'an, Shaanxi 710048, China

[c] Shaanxi Key Laboratory of Environmental Monitoring and Forewarning of Trace Pollutants, Shaanxi Province Environmental Monitoring Center, Xi'an, Shaanxi 710054, China

[d] Shaanxi Key Laboratory of Water Resources and Environment, Xi'an University of Technology, Xi'an, Shaanxi 710048, China

[e] CCTEG Xi'an Research Institute (Group) Co., Ltd., Xi'an, Shaanxi 710077, China

[f] Xi'an Modern Chemistry Research Institute, Xi'an, Shaanxi 710065, China

[g] School of Public Health and Health Management, Henan Medical College, Zhengzhou, Henan 451191, China

[h] Shaanxi Key Laboratory of Earth Surface System and Environmental Carrying Capacity, College of Urban and Environmental Sciences, Northwest University, Xi'an 710127, China

1. Materials and methods

Before the experiment, the strains were taken from the ultra-low temperature refrigerator and inoculated into 96-well plates. For 96-well plates, 120μl LB medium was added to each well, and then 20μl of thawed bacterial solution was added. The inoculated 96-well plates were cultured overnight at 37 °C (16-22h). A 1x M9 medium was used to dilute the bacterial solution at a ratio of 1:5, and the bacterial solution in the 96-well plate was transferred to the 384-well plate (with a certain arrangement order) by using the automatic pipetting workstation (German, Eppendorf AG, epMotion5075t). For the 384-well plate, 55 μl 1x M9 medium was added to each well, and then 5 μl bacterial solution was added and cultured at 37 °C for 5 h-6 h to make the optical density (OD) reach the early exponential growth stage (OD~0.2); after culture, 10 μl of different doses of disinfection by-products diluted with 1 x M9 medium were added to each well of the 384-well plate. To determine the effect of HMX-induced gene transcription level, the 384-well plate was placed in a microplate cell imaging system for simultaneous measurement of optical density (OD600, cell growth) and fluorescence readings (GFP level, Ex: 45 nm, Em: 528 nm). To develop a relatively rapid toxicity assessment method, the exposure time was selected as 2 h, and the reading was measured every 5 min. All tests were conducted in the dark and repeated three times.

All data were corrected by blank medium and promoterless bacterial controls (with and without toxicants), respectively. The gene expression alteration was called induction factor I ($I=P_e/P_c$), where P_e = $(GFP/OD)_{experiment}$ and P_c = $(GFP/OD)_{control}$. Then, the natural log of the I value (ln(I)) at every time point is compiled for further analysis(Gou and Gu, 2011). The toxicity values of chemicals were quantified by the TELI, and TELI values represent the magnitude of altered gene expression for each gene response to toxicant exposure for 2h (a test chemical is defined as toxic when the TELI value is >1.5) (Gou et al., 2014). The calculation method uses the following equation (Gou and Gu, 2011):

$$TELI_{gene}=\frac{\int_{t=0}^{t}\left(e^{|\ln I|}-e^{|\ln 1|}\right)dt}{\text{Exposure Time}} \quad (1)$$

$$TELI_{stress}=\frac{\sum_{i=1}^{n} wi\times(TELI_{genei})}{n} \quad (2)$$

$$TELI_{total} = \frac{\sum_{j=1}^{n} wj \times (TELI_{pathwayj})}{n} \quad (3)$$

where t (h) is the exposure time; i and j are the number of genes/stress pathway in the assay library; wi and wj are the weighting factors for the gene (i) and pathway (j); and in this study, all weighting factors were assigned a value of 1.

2. Figures

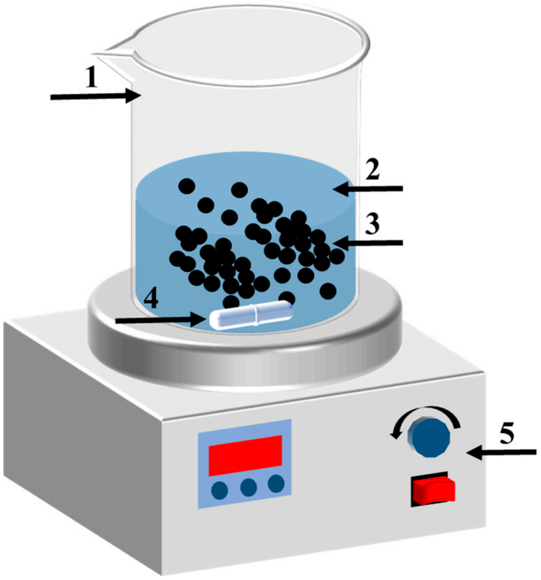

Figure.S1 Iron-carbon micro-electrolysis experimental setup diagram (1. Beaker; 2. HMX Wastewater; 3. Iron-Carbon Mixture; 4. Rotor; 5. Magnetic Stirrer)

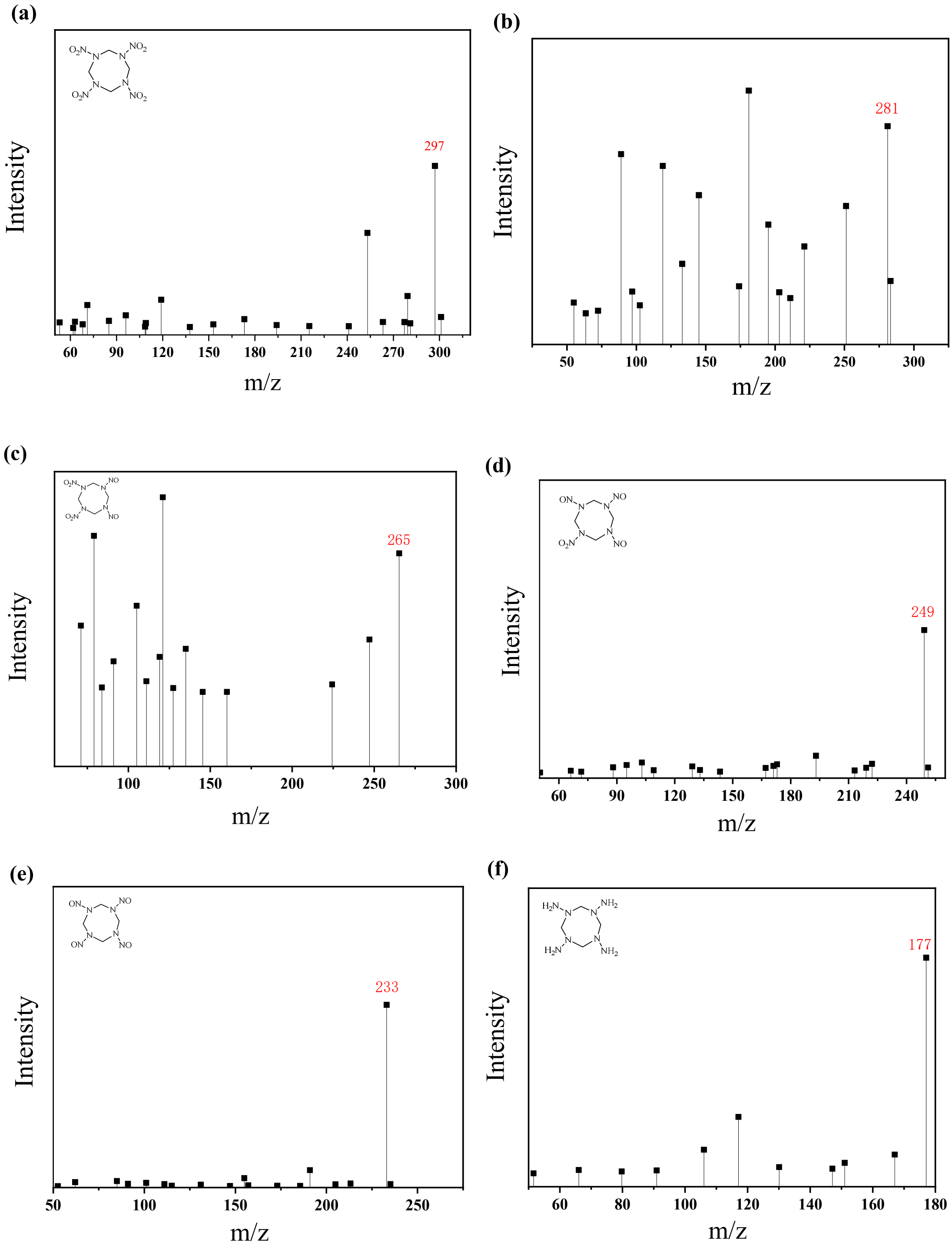

(a)
Intensity
m/z
60 90 120 150 180 210 240 270 300
297
(b)
Intensity
m/z
50 100 150 200 250 300
281
(c)
Intensity
m/z
100 150 200 250 300
265
(d)
Intensity
m/z
60 90 120 150 180 210 240
249
(e)
Intensity
m/z
50 100 150 200 250
233
(f)
Intensity
m/z
60 80 100 120 140 160 180
177

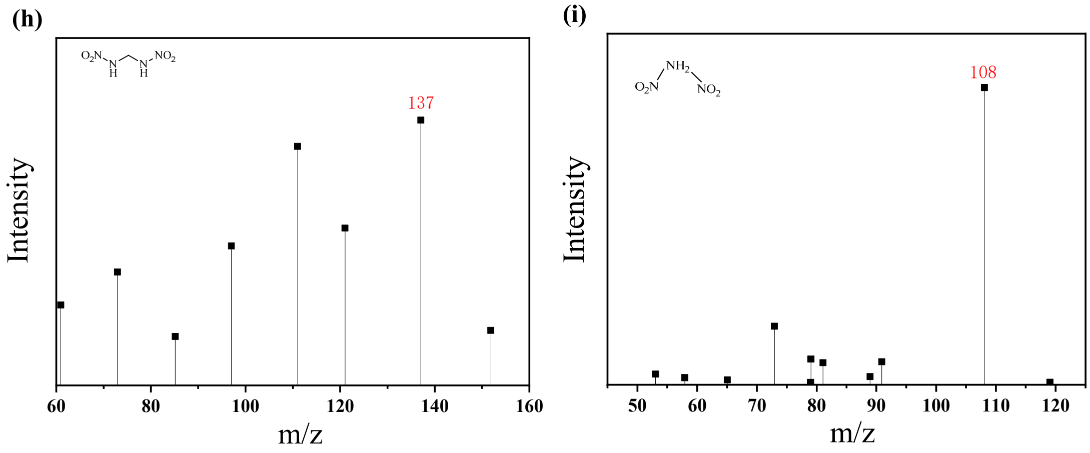

Figure.S2 Ion spectrum of HMX degradation intermediate products in positive ion mode

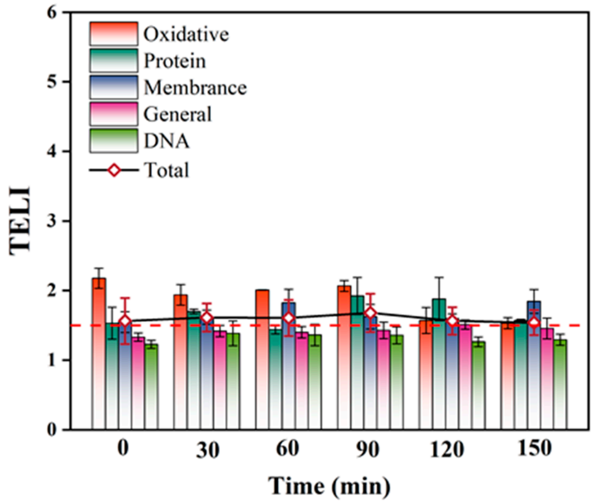

Figure S3. Changes in TELI-based toxicity profiles during Fe-C micro-electrolysis of HMX.

3. Tables

Table S1. Single-factor experimental design.

No.	Iron dosage (g/L)	Activated carbon （g/L）	Initial pH	Fe/C mass ratio
1	30.0	30.0	2.0	
2	30.0	30.0	3.0	
3	30.0	30.0	4.0	
4	30.0	30.0	5.0	
5	30.0	30.0	6.0	
6	30.0	30.0	7.0	
7	10.0	30.0	4.0	
8	20.0	30.0	4.0	
9	30.0	30.0	4.0	
10	40.0	30.0	4.0	
11	50.0	30.0	4.0	
12	60.0	30.0	4.0	
13	70.0	30.0	4.0	
14	70.0	140	4.0	0.5
15	70.0	116.7	4.0	0.6
16	70.0	87.5	4.0	0.8
17	70.0	77.8	4.0	0.9
18	70.0	70.0	4.0	1.0
19	70.0	63.6	4.0	1.1
20	70.0	58.3	4.0	1.2
21	70.0	53.8	4.0	1.3
22	70.0	50.0	4.0	1.4

Table S2. Stress gene library and its main functions

Category		Gene selected	Known functions
Genotoxic Stress		*uvrA, recE, clpB, rnt, recX, ada, dinB, mutT, nfo, ding, ftsk, recN, sbmC, ybfE, dnaQ, mutH, mutM, mutS, mutY, yjiW, mug, yebG, sulA, lexA, polB, recA, ssb, umuD, uvrD, ruvA, uvrC, uvrY, polA*	DNA strand breaks and cross-linking, alter superhelicity, oxidative DNA damage, base alkylation, inhibition of DNA synthesis and replication
Redox Stress	Oxidation	*soxS, soxR, oxyR, inaA, dps, ahpF, katG, sodA, ahpC, katE, ytfE, katE, sodB, sodC, trxA*	Increased levels of superoxides, increased levels of peroxides, any other conditions, which alter the redox potential of the cell. Genes response to oxidative stress
	Detoxification	*norR, fpr, tam, yeiG, yafN, yeaE, grxA, gst*	
Protein Stress		*clpB, ycgE, cueR, entC, grpE, dnaK, fepB, dnaJ, rpoD, lon,ybgl*	Denaturation, misfolding, cross-linking and alkylation of proteins, oxidation of individual amino acids and protein damage.
Membrane Stress	Energy stress	*sdhC, cyoA*	Perturbations of electron transport and exposure to uncoupling agents, which affect ATP levels in the cell.
	Drug resistance	*yedW, dacA, dacB, marR, sbmA, bacA, yhjX, emrE, sanA, emrA, marC, mdtK, yajR, fsr, cmr, mrcB, pbpG, ssrA, ompC*	Related to compound /chemical induced stress, most product located or functioned at inner or outer membrane
	Metabolism	*flgM*、*motA*、*cyoA*	
	Cell Membrane	*amiC, clsA,*	Related to cell membrane and phospholipid synthesis
General Stress	Cold shock	*cspA, cspB*	Temperature downshift
	Cell killing	*dinJ,*、*slyA*、*yeeV*、 *yfjG*	
	General stress	*uspA, otsB, ydgL, bolA, rpoE,*	Disturbance of the biochemical and biophysical homeostasis of the cell.

Table S3 Main degradation intermediates of HMX

Mid product	M+[H]	Possible molecular formulas	Molecular Structure	CAS
Original sample	297	$C_4H_8N_8O_8$	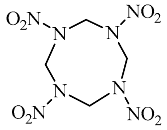	2691-41-0
II	281	$C_4H_8N_8O_7$	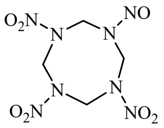	—
III	265	$C_4H_8N_8O_6$	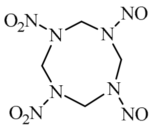	
IV	249	$C_4H_8N_8O_5$	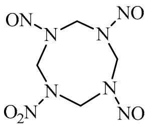	
V	233	$C_4H_8N_8O_4$	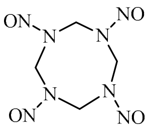	
VI	177	$C_4H_{16}N_8$	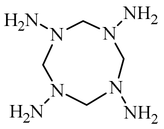	
VII	137	$CH_4N_4O_4$	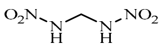	14168-44-6
VIII	108	HN_3O_4	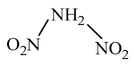	

Reference

1. Gou, N.; Gu, A.Z. A new transcriptional effect level index (TELI) for toxicogenomics-based toxicity assessment. *Environ. Sci. Technol.* **2011**, *45*, 5410–5417.
2. Gou, N.; Yuan, S.; Lan, J.; Gao, C.; Alshawabkeh, A.N.; Gu, A.Z. A quantitative toxicogenomics assay reveals the evolution and nature of toxicity during the transformation of environmental pollutants. *Environ. Sci. Technol.* **2014**, *48*, 8855–8863.
